# Endurance Exercise and Conjugated Linoleic Acid (CLA) Supplementation Up-Regulate CYP17A1 and Stimulate Testosterone Biosynthesis

**DOI:** 10.1371/journal.pone.0079686

**Published:** 2013-11-05

**Authors:** Rosario Barone, Filippo Macaluso, Patrizia Catanese, Antonella Marino Gammazza, Luigi Rizzuto, Paola Marozzi, Giuseppe Lo Giudice, Tomaso Stampone, Francesco Cappello, Giuseppe Morici, Giovanni Zummo, Felicia Farina, Valentina Di Felice

**Affiliations:** 1 Department of Experimental Biomedicine and Clinical Neurosciences (BioNeC), University of Palermo, Palermo, Italy; 2 Laboratory of Nuclear Medicine, Hospital “O.R. Villa Sofia–Cervello”, Palermo, Italy; 3 Laboratory of Clinical Pathology, Hospital “O.R. Villa Sofia–Cervello”, Palermo, Italy; Rutgers University, United States of America

## Abstract

A new role for fat supplements, in particular conjugated linoleic acid (CLA), has been delineated in steroidogenesis, although the underlying molecular mechanisms have not yet been elucidated. The aims of the present study were to identify the pathway stimulated by CLA supplementation using a cell culture model and to determine whether this same pathway is also stimulated *in vivo* by CLA supplementation associated with exercise. *In vitro*, Leydig tumour rat cells (R2C) supplemented with different concentrations of CLA exhibited increasing testosterone biosynthesis accompanied by increasing levels of CYP17A1 mRNA and protein. *In vivo*, trained mice showed an increase in free plasma testosterone and an up-regulation of CYP17A1 mRNA and protein. The effect of training on CYP17A1 expression and testosterone biosynthesis was significantly higher in the trained mice supplemented with CLA compared to the placebo. The results of the present study demonstrated that CLA stimulates testosterone biosynthesis via CYP17A1, and endurance training led to the synthesis of testosterone *in vivo* by inducing the overexpression of CYP17A1 mRNA and protein in the Leydig cells of the testis. This effect was enhanced by CLA supplementation. Therefore, CLA-associated physical activity may be used for its steroidogenic property in different fields, such as alimentary industry, human reproductive medicine, sport science, and anti-muscle wasting.

## Introduction

Fat supplements are nutritional ergogenic aids used by elite and recreational athletes; many sport magazines advise that their intake can improve endurance capacity, increase VO2max, reduce fat body mass, increase lean body mass, reduce muscle glycogen breakdown, improve metabolism, and prevent or reduce muscle damage and inflammatory responses [1]. These supplements include long-chain triacylglycerols, medium-chain triacylglycerols, fish oil, and conjugated linoleic acid (CLA) [2]. In the scientific community, a new role of dietary fat, particularly fat supplements, has been delineated in steroidogenesis, although the molecular mechanism has not yet been elucidated [2]. 

CLA, one of the commercially available fat supplements, refers to a group of positional and geometrical isomers of linoleic acid (cis-9, cis-12-octadecadienoic acid), an omega-6 essential fatty acid that exhibits various physiological effects including anti-adipogenic, anti-carcinogenic, and immune modulation effects [3]. The main isomers of CLA are cis-9, trans-11, trans-10, and cis-12 octadecaenoic acids (c9, t11-CLA and t10, c12-CLA) [3]. We have previously shown that CLA treatment stimulates testosterone biosynthesis in the rat Leydig tumour cell line (R2C), although the testosterone pathway stimulated by the CLA treatment was not identified [4]. High levels of testosterone may enhance muscle mass, which is important in sport science to increase maximal voluntary strength [5] and in cachexia and anti-aging therapies to stop muscle wasting [6–8]. Moreover, high levels of testosterone may improve endurance performance by increasing haemoglobin concentrations and haematocrit [9] as well as by increasing lactate transport through the enhancement of monocarboxylate transporter 1 and 4 in skeletal muscle [10].

To our knowledge, currently only two pathways have been shown to be responsible for the possible effect of CLA on testosterone biosynthesis. First, in adipocytes, perilipin and hormone-sensitive lipase (HSL) create a protective coat on the lipid droplet surface. Under stimulation, both proteins become hyperphosphorylated, and perilipin is displaced from the lipid droplet, allowing HSL to convert cholesteryl ester to free cholesterol. In Leydig cells, the same pathway may stimulate testosterone production under CLA treatment. Second, CLA may alter steroidogenesis by up-regulating specific genes encoding enzymes and transport proteins involved in the synthesis of testosterone, such as 17α-hydroxylase/17,20-lyase (CYP17A1), which converts progesterone into androstenedione. It has been demonstrated that a change in CYP17A1 expression may directly affect the level of testosterone [11,12].

We hypothesised that CLA supplementation may stimulate testosterone biosynthesis by two possible pathways: i) perilipin and HSL should become hyperphosphorylated, thereby allowing HSL to convert cholesteryl ester into free cholesterol to increase the hormone production; or ii) specific genes encoding enzymes and transport proteins involved in steroidogenesis should be up-regulated to promote testosterone production. The aims of the present study were, therefore, to identify the pathway stimulated by CLA supplementation using the rat Leydig tumour cell line and to determine whether the same pathway is affected by CLA supplementation in mice, as well as determine its association with exercise.

## Materials and Methods

### Cell cultures

R2C cells (cat. No. 89031606, ECACC, Health Protection Agency Culture Collections, Salisbury, United Kingdom) were cultured in M-199 medium (Invitrogen Corp., Carlsbad, CA, USA) supplemented with 15% horse serum (Invitrogen Corp), 2.5% foetal bovine serum (FBS; Invitrogen Corp.), and an antibiotic and antimycotic solution (100 U/mL penicillin, 100 µg/mL streptomycin, 0.25 µg/mL amphotericin B; Invitrogen Corp). Cells were incubated at 37°C in a humidified atmosphere with 5% CO_2_ and maintained in culture using standard techniques as previously described [4]. When cells reached approximately 80% confluence, they were treated for 48 h with different concentrations (0 to 7.5 µM) of CLA working solution; the CLA working solution was prepared by first dissolving Tonalin® FFA 80 (Cognis Nutrition and Health) in absolute ethanol and then in FBS containing 1% bovine serum albumin (BSA; Sigma - Aldrich, St. Louis, MO).

### Animal Experiments and Functional Tests

All animal experiments were approved by the committee on the ethics of animal experiments at the University of Palermo and adhered to the recommendations in the guide for the care and use of laboratory animals set by the NIH. Moreover, all experiments were performed in the Human Physiology Laboratory of the Department of Experimental Biomedicine and Clinical Neuroscience at the University of Palermo, which was formally authorised by Ministero della Sanità (Roma, Italy). Thirty-two male mice (BALB/cAnNHsd) were obtained from Harlan laboratories S.r.l. (Udine, Italy). The animals were kept at a constant 12:12 h light-dark cycle and had free access to food and water. Mice were randomly assigned to one of four groups (n = 8 per group): placebo sedentary (PLA-SED); CLA sedentary (CLA-SED); placebo trained (PLA-TR); or CLA trained (CLA-TR). The CLA groups (CLA-SED; CLA-TR) were gavaged with 35 µL per day (corresponding to the 0.5% of food ingested, approximately 4 g) Tonalin® FFA 80 food supplement containing CLA throughout the 6 week experimental period, while the placebo groups (PLA-SED; PLA-TR) were gavaged with 35 µL per day sunflower oil [13]. Mice were gavaged approximately 9:00 am, immediately before the training session. Trained groups (PLA-TR; CLA-TR) performed progressive running on the rotarod, which is a rotating cylinder on which the mice were forced to run to avoid falling down [14], for 6 weeks. Exercise training began at 3.2 m/min for 5 d/week; during week 1, the mice exercised for 15 min, and the time and speed were systematically increased until week 6, where they were training at 4.8 m/min for 60 min [15–17]. Sedentary mice (PLA-SED; CLA-SED) did not perform any supervised physical activity. Every 2 weeks, trained and sedentary mice were subjected to an endurance test on the rotarod, and the exercise time was measured until the mouse failed to run and fell from the cylinder. Grip tests were performed with a grip strength meter (47200 - Grip-Strength Meter, Ugo Basile, Italy) every 3 weeks to measure the strength of the forelegs. Briefly, this method is based on the measure of the grip force using a dynamometer while the mouse is being pulled by its tail [18]. Sedentary and trained mice were tested 5 times in succession without rest, and the best 3 results were averaged for each mouse. All mice were weighed every 3 weeks. Two days after the last exercise session, all the mice were sacrificed by cervical dislocation, and serum samples were taken and immediately stored in a -80°C freezer. The anterior muscle groups of the hindlimb (tibialis anterior, extensor hallucis longus, and extensor digitorum longus) and the testicles were dissected, weighed, and frozen in liquid nitrogen.

### Testosterone Biosynthesis

**Figure 1:**
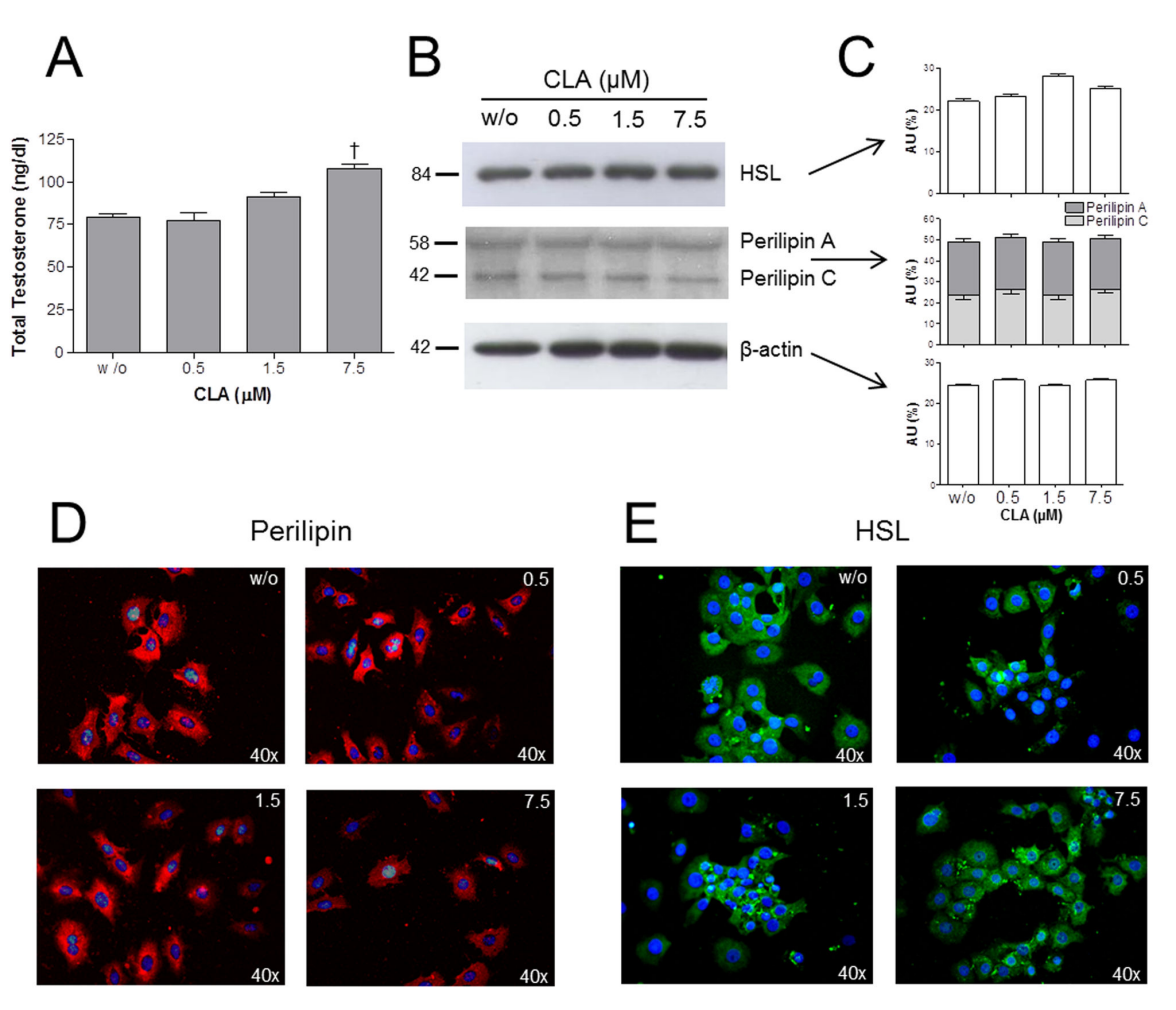
Effect of CLA supplementation on testosterone biosynthesis, perilipin A/C, and hormone sensitive lipase (HSL) levels in R2C cells. A. Total testosterone biosynthesis following treatment with different CLA concentrations (w/o, 0.5, 1.5 and 7.5 µM CLA). B. Representative western blots showing HSL (84 kDa), perilipin A/C (58 and 42 kDa, respectively), and ß-actin (42 kDa) expression following electrophoretic separation of protein extracts obtained from R2C cells treated with different concentrations of CLA. C. Relative amounts of HSL, ß-actin, perilipin A and C. D. Representative immunofluorescent images of perilipin A/C expression in R2C cells treated with different concentrations of CLA. E. Representative immunofluorescent images of HSL expression in R2C cells treated with different concentrations of CLA. † Significant difference compared to w/o and 0.5 µM CLA (P<0.01).

Testosterone measurements were performed in the laboratory of “Azienda Ospedaliera Villa Sofia–Cervello Palermo”. The total testosterone was measured using an Immulite 2000 (Siemens, Milan, Italy), according to the manufacturer’s instructions for the specific kit (Immunolite 2000 testosterone total), as described previously [4]. Free testosterone was measured by radioimmunoassay, according to the manufacturer’s instructions (DIAsource ImmunoAssays S.A., Belgium). Following treatment with 7.5 µM CLA working solution for 48 h, the biosynthesis of total testosterone secreted into the media had increased in the R2C cells (Figure 1A). The increase in testosterone level was significantly higher in the cells treated with 7.5 µM CLA working solution than in the control (w/o) and the 0.5 µM treated cells (P<0.01).

### Antibodies

The primary antibodies used in for the *in vitro* and *in vivo* experiments included anti-perilipin A/C (rabbit polyclonal antibody, ABR Affinity BioReagents, CO), anti-hormone sensitive lipase (PRS3965, rabbit polyclonal antibody, Sigma-Aldrich, Germany), anti-CYP17A1 (M-80, rabbit polyclonal antibody, Santa Cruz, CA), and anti-beta-actin (AC-40, mouse monoclonal antibody, Sigma-Aldrich, Germany). The dilution of antibodies was 1:1000 for western blotting and 1:50 for immunofluorescence staining.

### Western Blotting Analyses

Cells and half of the snap frozen testicles were lysed with lysis buffer (200 mM HEPES, 5 M NaCl, 10% Triton X-100, 0.5 M EDTA, 1 M DTT, 0.25 g Na-deoxycholate, 0.05 g SDS) supplemented with Protease Inhibitor Cocktail (Sigma-Aldrich, USA). Equal amounts of proteins (60 µg/lane) were measured using the Quant-iT TM protein assay kit (Invitrogen Molecular Probes, Italy) and a Qubit fluorometer according to manufacturer’s instructions and were separated using a 12% SDS-PAGE gel. The separated proteins were electrophoretically transferred to a polyvinylidene difluoride membrane (Hybond-PVDF, Amersham Biosciences, GE Healthcare, Little Chalfont, England) and incubated with a blocking solution containing 5% BSA (Sigma-Aldrich, USA) in Tris-Buffered Saline (20 mM Tris, 137 mM NaCl, pH 7.6) with 0.05% Tween-20 (T-TBS) for 1 h at RT. Blots were incubated in 0.5% BSA in T-TBS with primary antibodies diluted 1:1000 overnight at 4°C. Blots were washed in T-TBS and incubated for 1 h in 0.5% BSA in T-TBS with a secondary antibody (ECL™ anti-mouse/rabbit IgG, Horseradish Peroxidase linked whole antibody from sheep, GE Healthcare, UK). The final detection was performed using ECL Western Blotting Detection Reagent (Amersham Biosciences), according to the manufacturer’s instructions. Band analysis was performed with the ImageJ 1.41 software (National Institutes of Health, USA, http://rsb.info.nih.gov/ij). The phosphorylated state of the perilipin proteins was determined by visualisation of a delayed band in the western blotting analysis, as described by Servetnick et al. [19]. An antibody against the only phosphorylated form of the perilipins is not commercially available.

### Immunofluorescence Staining

This technique was performed as described previously [20,21]. Cells were fixed in ice-cold methanol. After fixation, the cells were incubated with unmasking solution (10 mM trisodium citrate, 0.05% Tween-20) for 10 min and treated with blocking solution (3% BSA in PBS) for 30 min at 24°C. Next, primary antibody was added, and the cells were incubated in a humidified chamber overnight at 4°C. The cells were then incubated for 1 h at RT with a conjugated secondary antibody (Anti-Rabbit IgG–TRITC antibody produced in goat; Anti-Rabbit IgG–FITC antibody produced in goat, Sigma-Aldrich) followed by 1a 5 min incubation with HOECHT33342 (Sigma-Aldrich) diluted 1:1000. Finally, the slides were mounted with cover slips, and images were taken immediately with a Leica DM5000 upright fluorescence microscope (Leica Microsystems, Heidelberg, Germany). 

### Real-Time quantitative PCR (qPCR)

The qPCR technique was previously described in Di Felice et al. [22]. Total cellular RNA was isolated from cell cultures and testicle samples using TRIzol® REAGENT (Sigma-Aldrich, USA), according to the manufacturer’s instructions. RNA (20 ng) was retro-transcribed using the ImProm-II Reverse Transciptase Kit (Promega Corporation) to obtain cDNA, which was amplified using the GoTaq qPCR Master Mix (Promega Corporation, USA). The mRNA levels were normalised to the levels obtained for hypoxanthine phosphoribosyltransferase 1 (HPRT1) and for beta-glucuronidase (GUSB). Changes in the transcript level were calculated using the 2-∆∆Ct method [22,23]. The cDNA was amplified using the primers indicated in Table 1. The fragments were purified using the Nucleospin PCR & Gel Clean Up Kit (MACHEREY-NAGEL GmbH & Co. KG, Düren, Germany) and were sequenced both forwards and backwards by the MWG Biotech Sequencing Service (MWG-Biotech, Inc., Edersberg, Germany). Sequences were then checked using the BLASTn Web Tool found on the NIH website (http://www.ncbi.nlm.nih.gov/BLAST/). 

**Table 1:** Forward and Reverse Primers used for qPCR.

	**Primer**	**Forward**	**Reverse**
**Rat**	***GUSB***	5’-ACCACCCCTACCACCTATATC-3’	5’-ATCCAGTAGTTCACCAGCCC-3’
	***HPRT1***	5’-TGTCATGAAGGAGATGGGAG-3’	5’-ATCCAGCAGGTCAGCAAAG-3’
	***CYP11A1***	5’-TTGCCTTTGAGTCCATCACC-3’	5’-CATGTTGAGCATGGGAACAC-3'
	***HSD3B1***	5’-CACCCTTTAACTGCCACTTG-3’	5’-ACTCCGAGGTTTTCTGCTTG-3’
	***CYP17A1***	5’-TGATCCAAAACTGACCGCC-3’	5’-TCCACCAGATTTCTGTCGCC-3’
	***STAR***	5’-CCTTGGGCATACTCAACAAC-3’	5’-GCACCACCTTACTTAGCAC-3’
**Mouse**	***GUSB***	5’-CAAGGGGTCAATAAGCACGA-3’	5’-TCTGAGTAGGGATAGTGGCT-3’
	***HPRT1***	5’-CAGCCCCAAAATGGTTAAGG-3’	5’-AAGTCTGGCCTGTATCCAAC-3’
	***CYP11A1***	5’-GGGCACTTTGGAGTCAGTTT-3’	5’-CGGTCTTTCTTCCAGGCATC-3’
	***HSD3B1***	5’-GCTGCTGCACAGGAATAAAG-3’	5’-GCCTGCTTCGTGACCATATT-3’
	***CYP17A1***	5’-ACACCTAATGCCAAGTTCCC-3’	5’-AGGCGAAGAGAATAGATGGG-3’
	***STAR***	5’-ACACCCCAAAGAAGGCATAG-3’	5’-GCTGAATCCCCCAAACTTCT-3’

### Statistical analyses

To assess the consistency of the results, each *in vitro* experiment was repeated at least 5 times. A one-way ANOVA followed by a Bonferroni post-hoc test for multiple comparisons was used as an appropriate analysis for the *in vitro* data. A two-way ANOVA (supplementation vs. exercise) for single or for repeated measurements followed by a Bonferroni post-hoc test was used for analysis of the *in vivo* data. All statistical analyses were performed using the GraphPad PrismTM 4.0 program (GraphPad Software Inc., San Diego, California, USA). All data are presented as the means ± SEM, and the level of statistical significance was set at p<0.05.

## Results

### In vitro experiments: R2C cell line

#### CLA Supplementation Does Not Affect Perilipin A/C and HSL Protein Levels

No differences in perilipin A/C and HSL expression were observed in the immunofluorescence experiments on R2C cells treated with different CLA concentrations compared to the control (Figure 1D and E). Furthermore, western blotting analysis did not reveal any differences in the amount of perilipin A/C and HSL proteins in R2C cells treated with different concentrations of CLA compared to the untreated cells. Moreover, there was no difference in the phosphorylation state of either isoform of perilipin (Figure 1B-C).

#### CLA Supplementation Up-Regulates CYP17A1 mRNA Expression

**Figure 2:**
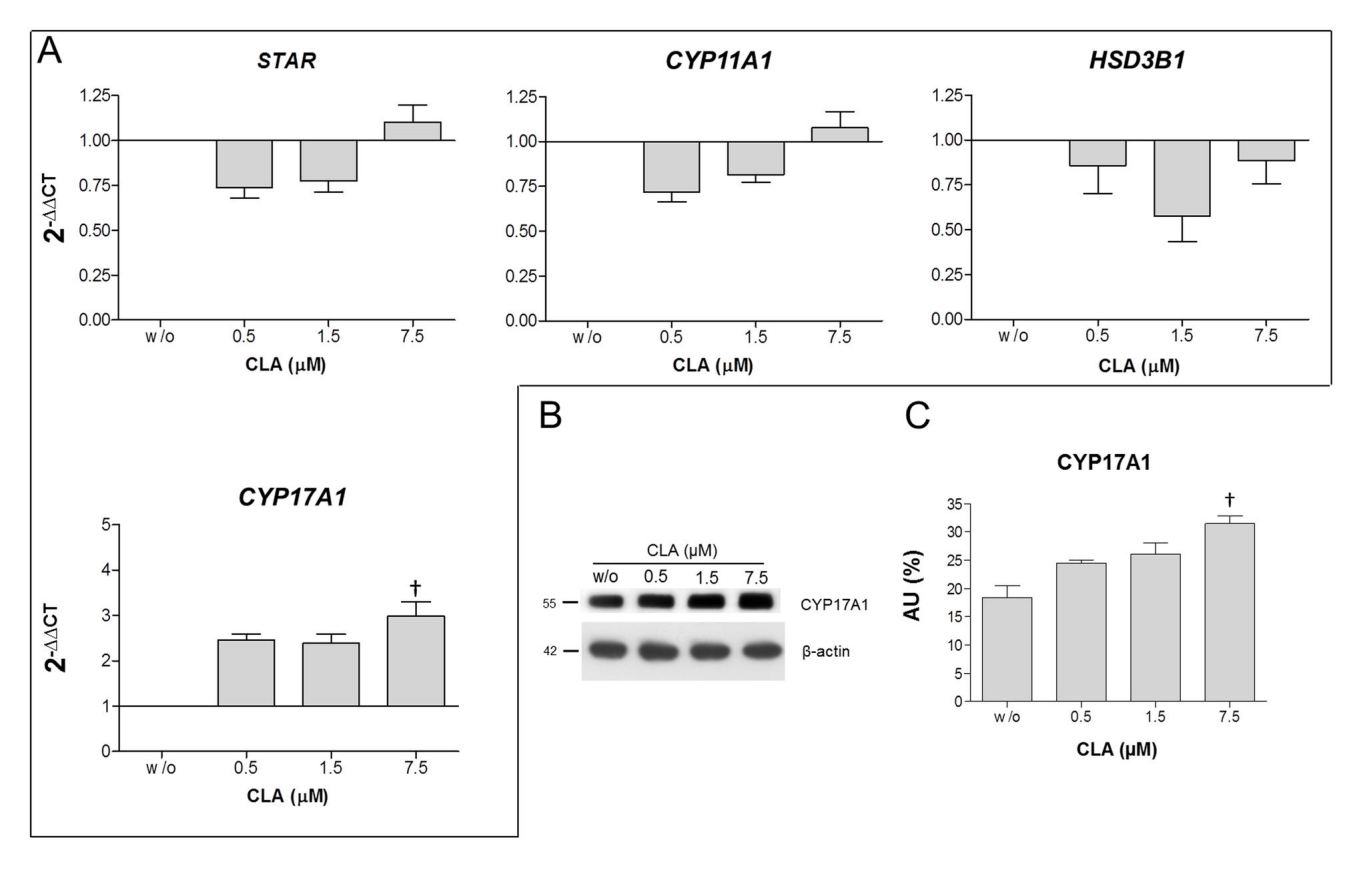
Effect of CLA supplementation on steroidogenic genes and proteins *in* *vitro*. A. Real-time PCR analysis of genes encoding steroidogenic enzymes from *in* *vitro* R2C cell extract. The graph shows the normalisation with the reference genes, according to the Livak Method (2-∆∆CT). B. Representative western blots showing CYP17A1 (55 kDa) and ß-actin (42 kDa) expression following electrophoretic separation of protein extracts obtained from R2C cells treated with different concentrations of CLA (w/o, 0.5, 1.5 and 7.5 µM CLA). C. Relative amounts of CYP17A1. † Significant difference compared to w/o CLA (P<0.01).

To investigate the effects of different concentrations of CLA on the testosterone biosynthesis pathway in R2C cells, the mRNA expression of specific genes encoding enzymes involved in the conversion of cholesterol to testosterone were measured using qPCR. Expression of STAR, CYP11A1, and HSD3B1 mRNA did not change following treatment with different concentrations of CLA. CYP17A1 mRNA expression in the R2C cells treated with 7.5 µM CLA working solution increased significantly compared to the control (P<0.01). The qPCR results of the R2C cells are summarised in Figure 2A.

#### CLA Supplementation Increases CYP17A1 Protein Level

Lysates of R2C cells treated with different CLA concentrations were analysed by western blotting analysis to examine if the increase in CYP17A1 mRNA expression translated into an increase in protein levels. The protein expression of CYP17A1 was significantly higher in R2C cells treated with 7.5 µM CLA working solution compared to the control (P<0.01) (Figure 2B and C).

### In vivo experiments

#### Exercise and CLA Increase Free Testosterone Levels

Two days after the last exercise session, the mice were sacrificed, and plasma collected to measure the levels of free testosterone. The endurance training induced a significant increase in free testosterone (PLA-SED: 0.35±0.02 pg/mL; PLA-TR: 20.50±3.54 pg/mL; P<0.01). The CLA supplementation did not induce any significant changes in free testosterone level in the sedentary mice (PLA-SED: 0.35±0.02 pg/ml; CLA-SED: 0.31±0.04 pg/mL), but in the trained mice, it stimulated a further increase in free testosterone (PLA-TR: 20.50±3.54 pg/mL; CLA-TR: 29.20±0.71 pg/mL; P<0.04).

#### CLA Supplementation Associated with Endurance Exercise Up-regulates CYP17A1 mRNA expression

**Figure 3:**
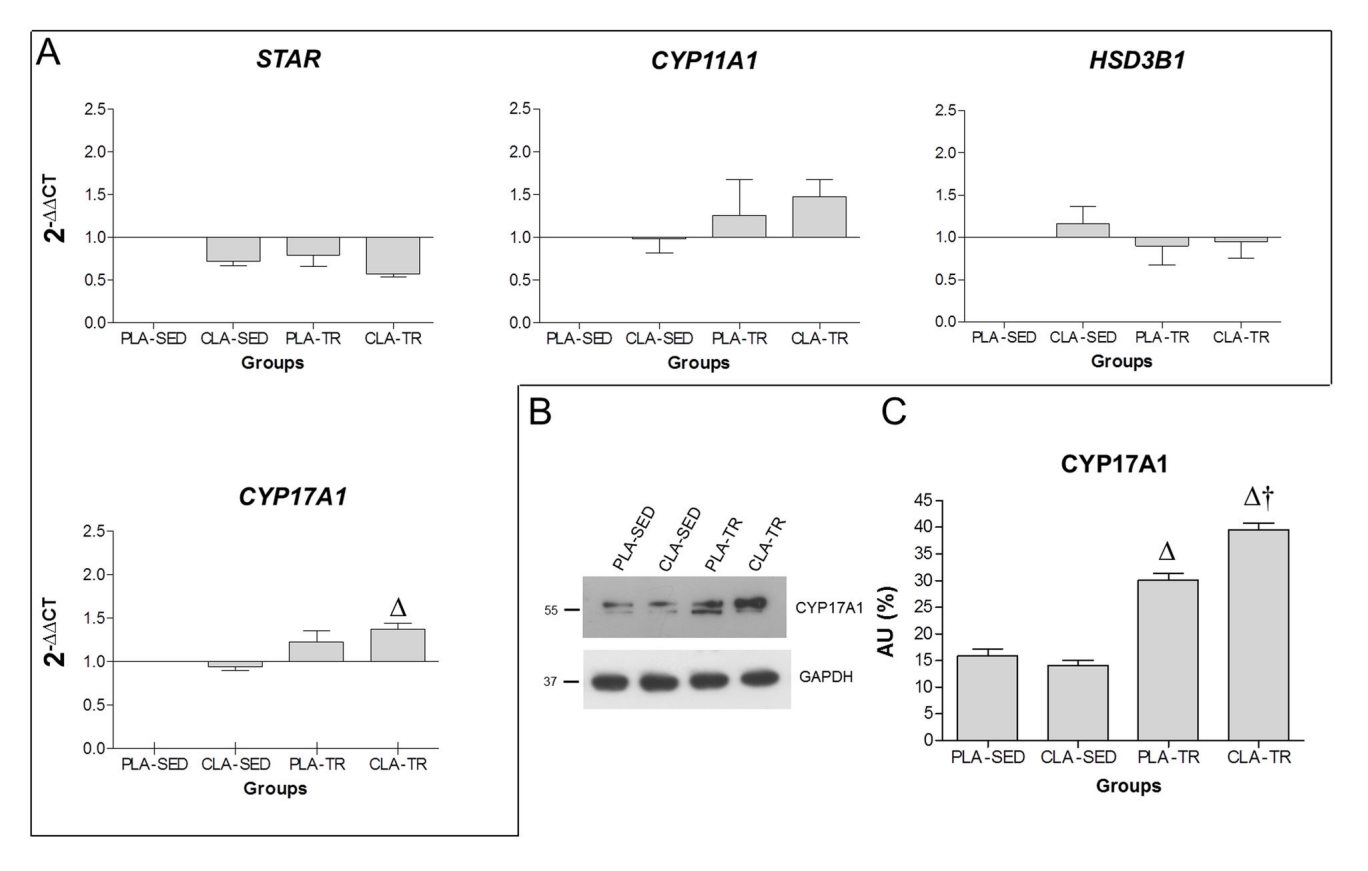
Effect of CLA supplementation on steroidogenic genes and proteins *in* *vivo*. A. Real-time PCR analysis of genes encoding steroidogenic enzymes from *in* *vivo* testicular extracts. The graph shows the normalisation with the reference genes, according to the Livak Method (2-∆∆CT). B. Representative western blots showing CYP17A1 (55 kDa) and ß-actin (42 kDa) expression following electrophoretic separation of protein extracts obtained from testicles. C. Relative amounts of CYP17A1. SED, sedentary; TR, trained; PLA, placebo; CLA, conjugated linoleic acid. ∆ significant difference compared to the PLA-SED and CLA-SED groups (P<0.01). † Significant difference compared to the PLA-TR group (P<0.01).

The mRNA extracted from the testicles of mice under the four different conditions were analysed using real-time quantitative qPCR to detect whether specific genes encoding enzymes involved in the conversion of cholesterol to testosterone were up-regulated (Figure 3A). Data analysis revealed an up-regulation of CYP17A1 mRNA in the CLA-TR group (P<0.01). The expression of STAR, CYP11A1, and HSD3B1 mRNA did not change under the different supplementation conditions (PLA and CLA) or in response to physical activity (SED and TR).

#### CLA Supplementation Induced a Greater Increase in CYP17A1 Protein Levels than Endurance Exercise Alone

The protein expression of CYP17A1 was significantly higher in both the trained groups (PLA-TR and CLA-TR) compared to the sedentary groups (PLA-SED and CLA-SED) (P<0.01). Moreover, CLA supplementation induced a further increase in CYP17A1 protein in the CLA-TR group compared to the PLA-TR group (P<0.01) (Figure 3B and C).

#### Functional Effect of Training and CLA Supplementation

**Figure 4:**
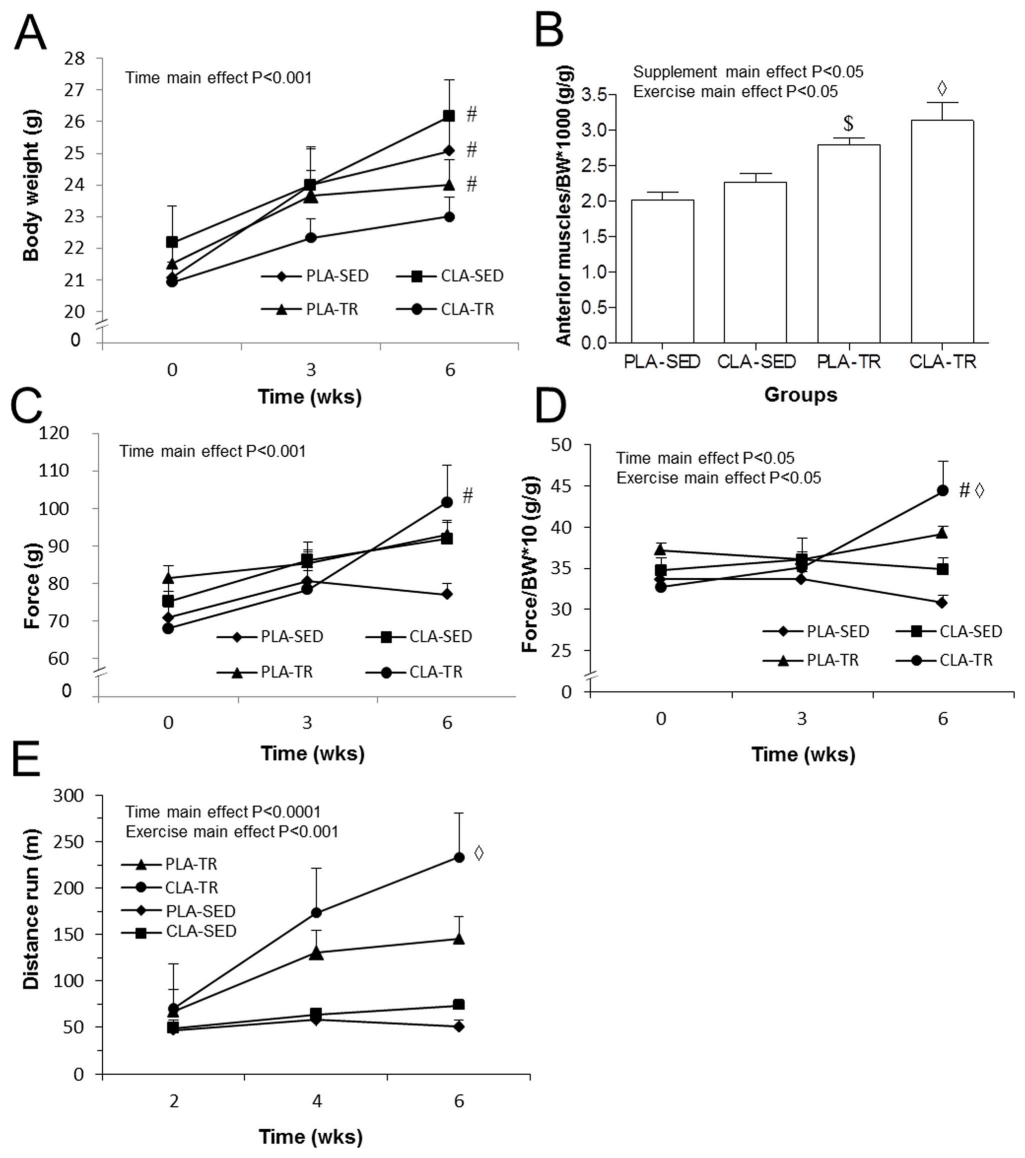
Effect of CLA supplementation on body weight, skeletal muscle, strength, and endurance performance *in* *vivo*. A. Changes in body weight over time. B. Weight of the hindlimb anterior muscle groups (tibialis anterior, extensor hallucis longus, and extensor digitorum longus) normalised for body weight. C. Strength of the forelimb. D. Strength of the forelimb normalised to the body weight. E. Distance ran during the endurance test. SED, sedentary; TR, trained; PLA, placebo; CLA, conjugated linoleic acid. # Significant difference compared to time point “0” (P<0.05). ◊ Significant difference compared to the CLA-SED group (P<0.05). $ Significant difference compared to the PLA-SED group (P<0.05).

Body weight, strength, and endurance performance were measured to study the functional effects of endogenous testosterone stimulation by CLA supplementation and exercise. Moreover, to evaluate hypertrophy, the anterior muscle groups of the hindlimb were excised and weighed following euthanasia. Due to normal growth during the 6 weeks of experimentation, the mice had a physiological increase in body weight. The increased body weight was significant in all groups (P<0.05) except in the CLA-TR group. No significant difference was observed among the groups (Figure 4A). The training exercise and the CLA supplementation had an anabolic effect and increased the muscle mass of the hindlimbs (Figure 4B). The CLA-TR and PLA-TR groups presented with heavier anterior hindlimb muscle groups, after normalisation for body weight, compared to the CLA-SED and PLA-SED groups (P<0.05), respectively. When the strength data were not normalised for body weight, no significant difference was observed among the groups because of the large variability between the mice; however, only the CLA-TR group showed a significant increase after 6 weeks (P<0.05). When the strength data were normalised for body weight, the CLA-TR group had greater strength gains compared to the CLA-SED group (P<0.05) after 6 weeks. The strength data are summarised in Figure 4C and D. The CLA-TR group also showed an improvement in the endurance performance test compared to the CLA-SED group after 6 weeks (Figure 4E).

## Discussion

The main finding of this study was the identification of a novel pathway that stimulates the biosynthesis of testosterone both *in vitro* and *in vivo* following CLA supplementation. In R2C cells supplemented with different concentrations of CLA, the increase in testosterone biosynthesis occurred along with the up-regulation of CYP17A1 mRNA and protein expression. *In vivo*, trained mice exhibited an increase in plasma free testosterone and the up-regulation of CYP17A1 gene expression, as well as the overexpression of the CYP17A1 protein in the testicles. This effect of training was more evident in the trained mice supplemented with CLA.

At the beginning of the study, two possible pathways were identified as possible targets of CLA. The first pathway identified was based on the lipolytic effect of CLA on adipocytes, which appears to be mediated by an increase in perilipin A protein levels [24]. In steroidogenic cells, there are two isoforms of perilipin, namely the A and C isoforms [19]. In normal Leydig cells, testosterone production is dependent upon cholesteryl esterase activation by 3’-5’-cyclic adenosine monophosphate (cAMP) [19], and lipid hydrolysis depends on the displacement of the phosphorylated perilipin A/C proteins from the membrane of the lipid droplet [25]. The results showed that different CLA concentrations did not affect HSL, perilipin A/C expression, or their phosphorylation status for 48 h in R2C cells. CLA supplementation could possibly modulate its effects by affecting different pathways depending on the tissue and cell type analysed.

The second pathway investigated was based on the hypothesis that CLA may alter steroidogenesis by up- and down-regulating specific genes encoding enzymes and transport proteins involved in the conversion of cholesterol into testosterone. There are many steroidogenic enzymes involved in testosterone biosynthesis, including steroidogenic acute regulatory protein (STAR), which transfers cholesterol to the inner membrane of mitochondria, cholesterol side chain cleavage enzyme (CYP11A1), which converts cholesterol into pregnenolone within the mitochondria, 3ß-hydroxysteroid dehydrogenase (HSD3B), which converts pregnenolone into progesterone, CYP17A1, which converts progesterone into androstenedione, and 17ß-hydroxysteroid dehydrogenase (HSD17B3), which converts androstenedione into testosterone [26]. The mRNA levels of the genes encoding these enzymes were measured using qPCR, and this analysis showed that the expression of STAR, CYP11A1, and HSD3B1 mRNA did not change under different CLA concentrations; however, CYP17A1 mRNA expression increased significantly in R2C cells treated with 7.5 µM CLA. The increase in CYP17A1 mRNA translated into an increase in the encoded protein; in fact, CYP17A1 protein expression was significantly higher in R2C cells treated with 7.5 µM CLA. The CYP17A1, CYP11A1, and STAR proteins are involved in the rate-limiting step of the testosterone biosynthesis pathway, which should have a rapid and marked effect on testosterone production when stimulated [27–30]. Moreover, CYP17A1 is one the main proteins studied in Leydig cells during toxicology experiments investigating the effects of drugs or compounds on steroidogenesis. It has been demonstrated that a change in the expression of CYP17A1 mRNA and protein may directly affect the level of testosterone [11,12]. Our results led us to believe that we have determined the pathway through which CLA affects steroidogenesis. However, future studies are needed to explain how CYP17A1 is affected by CLA.

In the literature, CLA supplementation appears to be associated with a reduction in body weight, an increase in lean body mass, and a reduction in body fat mass [3]; although, the most interesting results were obtained when CLA supplementation was associated with regular and supervised physical activity [2]. It appears that exercise is needed for CLA to induce its effects. The 6 weeks of endurance training induced an increase in free plasma testosterone and CYP17A1 enzyme in the testicles of mice. Interestingly, CLA supplementation induced a further increase in free plasma testosterone and CYP17A1 enzyme in the trained animals, while the sedentary animals supplemented with CLA or the placebo did not show any results indicative of an increase in steroidogenesis. Although it is known that exercise increases the amount of testosterone in circulation, it is unknown which protein mediates this effect. Our results indicate that the CYP17A1 enzyme may be the protein that plays a key role in steroidogenesis mediated by endurance exercise. It has previously been reported that increased plasma testosterone levels in males during exercise is luteinising hormone-independent, and instead depends on the levels of blood lactate [27,31]. The effect of lactate on the release of testosterone by the testicular tissue is modulated by the production of cAMP. Lin et al. [27] reported that physiological lactate concentrations did not change the expression of CYP11A1 and STAR but might increase the activity of CYP11A1 to stimulate testosterone production in rat Leydig cells. With the exception of this mechanism, no other mechanism or pathway has been proposed to explain the increase in testosterone levels after endurance exercise. Interestingly, Stojkov et al. [32] reported the inhibition of steroidogenesis in adult rats immobilised for 2 h, along with a down-regulation of SCARB1 (scavenger receptor class B), CYP11A1, CYP17A1, and HSD17B3 expression in the Leydig cells. Our *in vivo* data, which confirmed the *in vitro* data, showed that CLA supplementation may induce a further up-regulation of CYP17A1, resulting in an increase in circulating testosterone levels. Though the blood lactate concentrations were not measured in the present study, we maintain that the previously cited mechanism does not explain the present results as CLA supplementation appears to reduce blood lactate after 40 min of running in mice [33], and the levels of testosterone were measured 48 h after the last running session.

The effects of exogenous testosterone on skeletal muscle are well known [34], and consequently, the role of endogenous testosterone on contractile protein synthesis and satellite cells is well established [35-38]. Endurance exercise for 6 weeks induced hypertrophy, as the anterior hindlimb muscle groups of both trained groups were heavier than the sedentary groups; although a main effect of supplementation was detected, the hypertrophy recorded in the mice treated with CLA was not significantly higher than the placebo, most likely due to the reduced number of mice. The smaller increase in body weight in the CLA-TR group could be interpreted as a reduction in body fat mass. Although direct measures of lean and body fat mass were not performed in the present study, we have previously reported significant hypertrophy of the anterior tibialis in mice trained and supplemented with CLA for 6 weeks, using the same protocol as the present study [17]. Previous studies on CLA supplementation associated with physical activity in rodents have reported a reduction in body fat mass. In particular, Bhattacharya et al. [39] reported that the combination of dietary CLA and exercise decreased fat mass and increased lean mass in mice fed a high-fat diet, and these effects may be related in part to decreased serum leptin levels and exercise-induced increases in oxygen consumption and energy expenditure. One previous study reported that CLA has no effect on lean body mass or body fat mass, showing that there was no interaction between CLA treatment and physical activity in adult rats [40]. These differences seem to be mediated by an age factor; in fact, CLA diets were shown to reduce adipose tissue and increase muscle mass in growing animals. However, these effects are limited to adult animals. This is the first investigation into the changes in strength in mice supplemented with CLA, but the results in humans indicate that CLA supplementation does not enhance strength gains in bodybuilders [41] and in physically active subjects [4]. Colakoglu et al. [42] showed that CLA supplementation induced a small improvement in endurance performance in women trained with an aerobic training protocol. In our study, the trained mice supplemented with CLA showed an increase in endurance exercise capacity with respect to the trained mice supplemented with the placebo. The CLA supplementation could improve the endurance performance in three ways: i) higher testosterone may increase lactate transport through the enhancement of monocarboxylate transporter 1 and 4 in skeletal muscle [10]; ii) higher testosterone levels may induce an increase in haemoglobin concentration and haematocrit [9]; or iii) high fat oxidation and low consumption of stored liver glycogen as substrates for energy metabolism [33,43]. Neither of these proposed mechanisms can be excluded or categorically favoured.

 In conclusion, the results of the present study show that Leydig cells respond to CLA treatment or supplementation, enhancing testosterone biosynthesis by increasing the expression of CYP17A1. Because of the endogenous effect of CLA supplementation on testosterone production, this food supplement, in association with physical activity, may be used as an ergogenic aid in different fields of interest, including in sport science to enhance the positive effect of training on muscle mass and performance, in cachexia and anti-aging therapy to reduce the muscle wasting, in the alimentary industry to increase the number of progeny for butchery butcheries, and in human reproductive medicine to help aid in spontaneous conception or to increase the chance of conception with assisted reproductive treatment. Because this study was performed in mice, the results reveal its limitation on species generalisation. Thus, future research in other animal models and humans are needed to confirm these findings.
